# Pyorrhœa Alveolaris

**Published:** 1894-03

**Authors:** 


### Dental Department.

[The JOURNAL is in receipt of the following contribution from an experienced dentist of Austin. As dentistry is now recognized as a branch of medicine, and dentists affiliate with the regular medical profession, and are represented by membership and a Section in the American Medical Association, we will open a department of dentistry, and invite contributions:]

*Editor Texas Medical Journal:*

I hand you a few lines on a very common and very troublesome disease, one which the physician, especially in the country, may be called on to treat:

"PYORRHŒA ALVEOLARIS."

"Pyorrhœa alveolaris," or as it is commonly called, "Riggs' disease," is an inflammation of the peridental membrane which surrounds the teeth, causing this delicate structure to suppurate and waste away. The teeth affected become loose and fall out of the mouth, or they are easily extracted. The disease may confine itself to one, two, or three teeth; very often, however, the entire row becomes involved. The patient may not know of its presence until it has progressed so far as to be beyond the power to arrest it. Indeed, until the last few years, the dental profession seem to have known very little about this troublesome affection, but we are glad to note that it is now receiving thorough investigation. Recent researches have developed many new facts thereto. It has been noticed, that the teeth of the young persons, that is, those under twenty-five years of age, are rarely affected; and it is further claimed, by those who have made a careful study of the disease, that it is of constitutional origin, and believed to exist more commonly in those who are troubled with lithæmia, which disease causes an excess of the urates in blood. And there is no doubt that when enamel is roughened from chemical abrasion, the urates are precipitated from the blood and saliva, and find lodgment on the rough enamel surface. When a nucleus is once formed, the deposit continues until the gum is forced away, and the salt continues beneath, as it did above the gum margins, until the alveolar process is encroached upon and wastes away, and the teeth lose their support.

Pyorrhœa may exist without any deposit being found on the teeth, and it has been known to exist in cases wherein no pockets were discernible, the only evidence of its ravages being a characteristic shrinkage of the gum, a shriveling of the gum at and above the margin, but no recession having occurred. The gums are anæmic and thin, and the loss of part of the alveolar border has caused the gum to sink down, so that a crease or wrinkle can be seen along the line of the loss of the hard tissue under them. There is a slight loosening of the teeth perceptible to the fingers, but there are no concretions under the gums. It is evident that the tissues above alluded to are not affected until their nutrition is disturbed; and as digistion is the bugbear of all such disturbances, we are to look in that direction for remedial agents. We are confirmed in this opinion by the fact that we can control this trouble, in a measure, by treatment and diet adapted to a condition of excess of uric acid in the system. The lesions are favorably affected by dietetic treatment.

Local treatment should be of such character as to first remove the deposit; second, wash out the pockets with an agent which will effectually destroy germs, bacteria and microbes. For this purpose we would advise the use of peroxide of hydrogen, then with 25 per cent. sulphuric acid, introduced with a stick, immediately followed with a solution of soda bicarbonate, when the effervescence shall have ceased; the pockets must again be washed with warm water, and thirdly, fill the pockets with a paste of the sulphate of quinine. This last is intended as an antiseptic and stimulant. F. S. C.

